# Temperature Monitoring During Ecmo: An in Vitro Study

**DOI:** 10.1186/2197-425X-3-S1-A506

**Published:** 2015-10-01

**Authors:** F Mojoli, S Bianzina, L Caneva, G Tavazzi, S Mongodi, M Pozzi, A Orlando, A Braschi

**Affiliations:** Anesthesia and Intensive Care, Fondazione IRCCS Policlinico S. Matteo, University of Pavia, Pavia, Italy

## Introduction

The need of heat exchanger in the ECMO circuit is controversial. Moreover, how to monitor patient central temperature during extracorporeal support is still not clear, but potentially useful for the detection of “unexpressed” fever, eventually related to septic complications.

## Objectives

We conducted two in vitro experiments to estimate ECMO heat dispersion and obtain clinical information regarding patient central temperature during extracorporeal support.

## Methods

Experiment A. We analyzed heat dispersion of an ECMO circuit (Maquet Rotaflow PLS System) at 36 combinations of blood flow (BF 1-3-5 L/min), gas flow (GF 0-5-10 L/min) and set temperature (T set 36-37-38-39 °C). in any condition, heat dispersion was considered equal to the power (Watts) generated by the heat exchanger at the steady state, defined as stable temperature throughout the circuit.

Experiment B. By a reservoir bag, we connected two circuits (pump+oxygenator+heat exchanger), one simulating the patient and the other the ECMO circuit. Patient and ECMO circuit T set ranged 36-39 °C and 35-39 °C, respectively, for overall 63 conditions; ΔT was patient - ECMO circuit T set difference. the power generated by the two heat exchangers (Watts, W) was recorded at constant patient BF (5 L/min) and ECMO BF and GF (3 L/min each).

## Results

**Figure 1:**
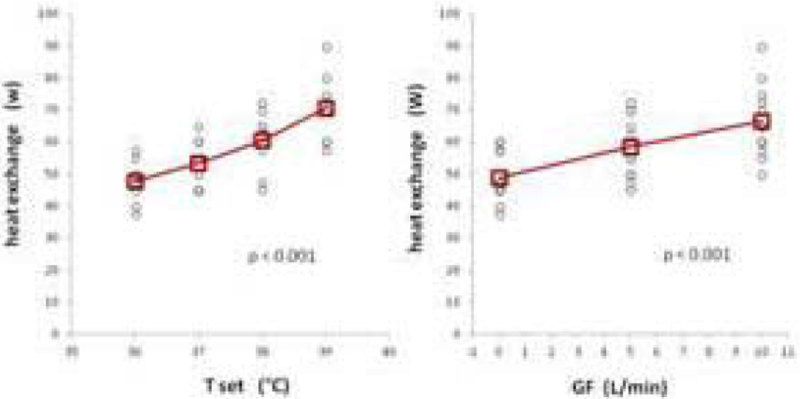
Figure 1

A) Overall, BF was 3.0 ± 1.7 L/min, GF 5.0 ± 4.1 L/min, pump rate 1299 ± 615 rpm, T set 37.5 ± 1.1 °C and circuit heat dispersion 58 ± 12 W (range 37.5-90), corresponding to a supposed patient metabolic consumption of 1196 ± 246 Kcal/die (range 774-1857) in case of heat exchanger absence or inactivation. It was affected by GF and T (p < 0.001), but not by BF (p = 0.9) (Figure 1).

**Figure 2:**
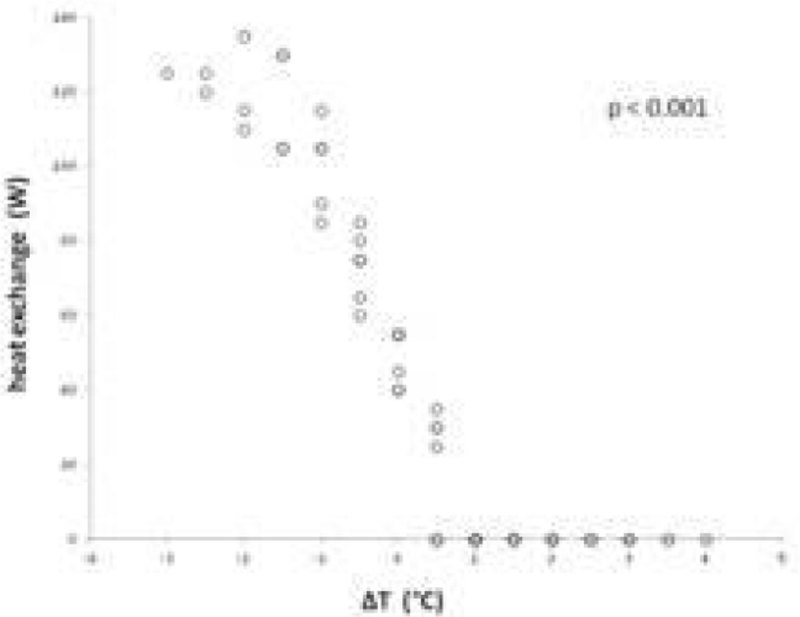
Figure 2

B) For ΔT > 0 heat exchanger did not generate energy, while for ΔT < 0 it supplied energy proportional to ΔT (p < 0.001). For ΔT = 0, heat exchanger power was 49 ± 7 W (range 40-55) (Figure 2).

## Conclusions

ECMO heat dispersion depends on GF and temperature, but not on BF. Therefore, heat exchanger should be considered also during low BF/high GF ECCO_2_R. T set on heat exchanger is well matched with patient central temperature when its power is in the 40-55 W range, whereas lower power values may be associated to patient “unexpressed” fever.

